# Two cadmium coordination polymers with bridging acetate and phenyl­enedi­amine ligands that exhibit two-dimensional layered structures

**DOI:** 10.1107/S2056989016017382

**Published:** 2016-11-04

**Authors:** David K. Geiger, Dylan E. Parsons, Bracco A. Pagano

**Affiliations:** a Department of Chemistry, SUNY-College at Geneseo, Geneseo, NY 14454, USA

**Keywords:** crystal structure, coordination polymer, metal organic framework, cadmium complex

## Abstract

Poly[tetra-μ_2_-acetato-κ^8^ *O*:*O*′-bis­(μ_2_-benzene-1,2-di­amine-κ^2^ *N*:*N*′)dicadmium], [Cd_2_(CH_3_COO)_4_(C_6_H_8_N_2_)_2_]_*n*_, (I), and poly[[(μ_2_-acetato-κ^2^ *O*:*O*′)(acetato-κ^2^ *O*,*O*′)(μ_2_-benzene-1,3-di­amine-κ^2^ *N*:*N*′)cadmium] hemihydrate], {[Cd(CH_3_COO)_2_(C_6_H_8_N_2_)]·0.5H_2_O}_*n*_, (II), have two-dimensional polymeric structures in which monomeric units are joined by bridging acetate and benzenedi­amine ligands. Each of the Cd^II^ ions has an O_4_N_2_ coordination environment. The coordination geometries of the symmetry-independent Cd^II^ ions are distorted octa­hedral and distorted trigonal anti­prismatic in (I) and distorted anti­prismatic in (II). Both compounds exhibit an intra­layer hydrogen-bonding network. In addition, the water of hydration in (II) is involved in inter­layer hydrogen bonding.

## Abstract 2

Two cadmium coordination polymers have two-dimensional polymeric structures in which monomeric units are joined by bridging acetate and benzenedi­amine ligands. Each of the Cd^II^ ions has an O_4_N_2_ coordination environment.

## Chemical context   

Cd^II^ is able to substitute for Zn^II^ in the active sites of zinc-containing enzymes and to inter­fere with the metabolism of Ca^II^, which are the primary reasons for its toxicity (Borsari, 2014 ▸). In addition, the substitution of Cd^II^ for spectroscopically silent Zn^II^ provides a means of exploring zinc-containing biomolecules using ^111^Cd and ^113^Cd NMR spectroscopies (Kimblin & Parkin, 1996 ▸; Henehan *et al.*, 1993 ▸; Jalilehvand *et al.*, 2009 ▸, 2012 ▸). Thus, the coordination chemistry of cadmium is of inter­est.

Metal–organic frameworks (MOFs) have received much attention because of their many potential applications including gas storage, catalysis, chemical sensors and mol­ecular separation (Dey *et al.*, 2014 ▸; Kreno *et al.*, 2012 ▸; Farha & Hupp, 2010 ▸). Our previous efforts in the area of coordination polymers have focused on compounds based on phenyl­enedi­amine and acetate ligands incorporating Zn^II^ (Geiger & Parsons, 2014 ▸) and Pb^II^ (Geiger *et al.*, 2014 ▸). We have extended this work to include Cd and report the structural analyses of two Cd compounds herein. Although acetate ligands adopt a myriad of different metal-binding modes, only the μ_2_-acetato-κ^2^ *O*:*O*′ mode is observed in (I). Both acetato-κ^2^ *O*,*O*′ and μ_2_-acetato-κ^2^ *O*:*O*′ modes are found in (II).

Numerous examples of structures with benzene-1,2-di­amines exhibiting monodentate and/or bidentate coordination modes have been reported (Narayanan & Bhadbhade, 1996 ▸; Ovalle-Marroquín *et al.*, 2002 ▸; Ariyananda & Norman, 2005 ▸; Chen *et al.*, 2006 ▸; Maxcy *et al.*, 2000 ▸; Qian *et al.*, 2007 ▸; Dickman, 2000 ▸; Mei *et al.*, 2009 ▸; Djebli *et al.*, 2012 ▸; Zick & Geiger, 2016 ▸; Geiger *et al.*, 2014 ▸; Geiger & Parsons, 2014 ▸; Geiger, 2012 ▸). Examples of benzene-1,4-di­amine metal-complex structures have also been reported (Batten *et al.*, 2001 ▸; Faizi & Prisyazhnaya, 2015 ▸). Few examples of bridging benzene-1,2-di­amine-κ^2^ *N*:*N*′ (Liang & Qu, 2008 ▸; Duff, 1968 ▸), 1,3-di­amine-κ^2^ *N*:*N*′ (Chemli *et al.*, 2013 ▸), or benzene-1,4-di­amine-κ^2^ *N*:*N*′ (Liu *et al.*, 2012 ▸) ligands have been reported. Compounds (I) and (II) are two new examples of coordination polymers in which benzenedi­amine ligands bridge two metal atoms.
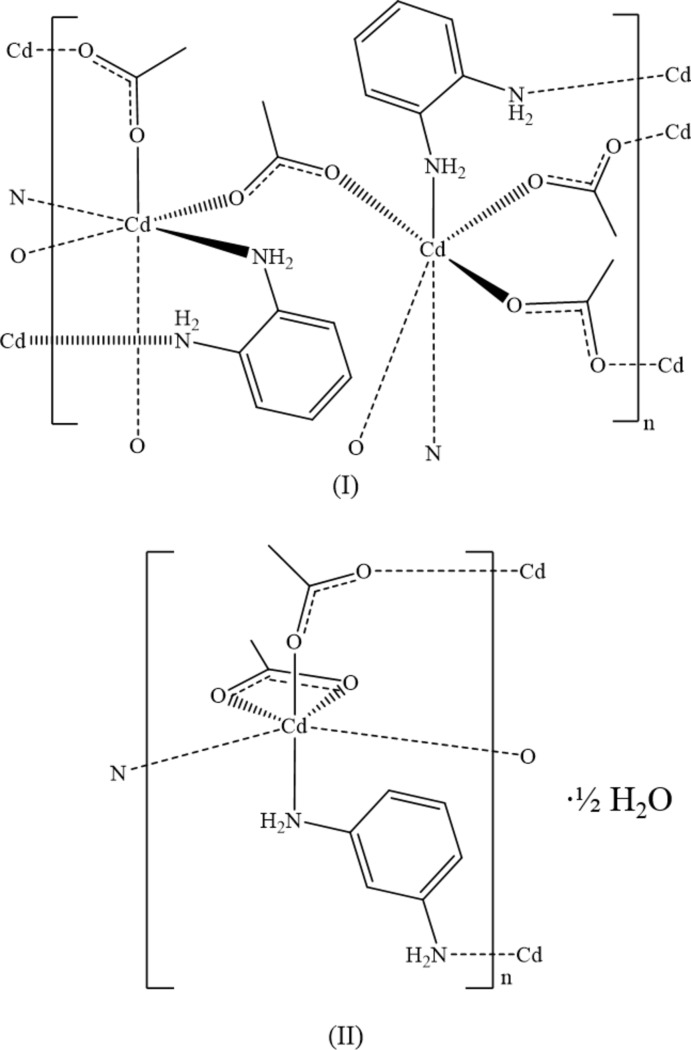


## Structural commentary   

**Figure 1:**
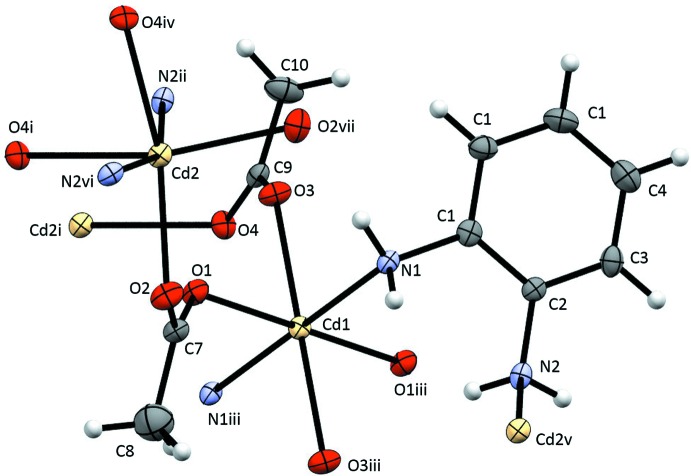
The atom-labeling scheme for (I). Anisotropic displacement parameters for non-H atoms are drawn at the 30% probability level. [Symmetry codes: (i) −*x* + 1, −*y* + 3, −*z* + 2; (ii) *x*, *y* + 1, *z*; (iii) −*x* + 1, −*y* + 2, −*z* + 2; (iv) *x*, −*y* + 3, *z* − 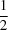; (v) *x*, *y* − 1, *z*; (vi) −*x* + 1, *y* + 1, −*z* + 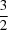; (vii) −*x* + 1, *y*, −*z* + 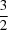.]

**Figure 2:**
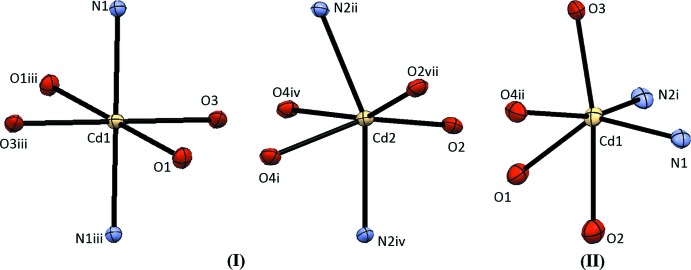
Representations of the Cd^II^ coordination environments observed in (I) and (II). Symmetry identifiers are those used in Figs. 1 ▸ and 3 ▸.

As shown in Fig. 1 ▸, (I) has two symmetry-independent Cd^II^ ions. Cd1 sits on a crystallographically imposed inversion center and Cd2 resides on a crystallographically imposed twofold rotation axis. Each of the Cd^II^ ions exhibits an O_4_N_2_ coordination sphere composed of four bridging κ^2^ *O*:*O*′ acetate ligands and two bridging κ^2^ *N*:*N*′ benzene-1,2-di­amine ligands. For the coordination sphere of Cd1, the twist angles (Muetterties & Guggenberger, 1974 ▸; Dymock & Palenik, 1975 ▸) defined employing the triangular face centroids N1O1O3 and N1^iii^O3^iii^O1^iii^ (see Fig. 2 ▸) are 52.26 (12), 66.27 (15) and 56.47 (9)°, giving an average value of 60 (5)°. Perfect O_h_ or D_3d_ trigonal anti­prismatic structures have a twist angle of 60°, whereas a D_3h_ trigonal prismatic structure has a twist angle of 0°. The coordination sphere of Cd2 exhibits twist angles of 35.49 (8), 45.92 (17) and 45.92 (17)° [average 42 (6)°] using opposite triangular faces O2O4^i^N2^iv^ and N2^ii^O4^iv^O2^vii^ (see Fig. 2 ▸). The coordination geometry is best described as distorted octa­hedral with the two nitro­gen donor atoms *trans* for Cd1 and distorted trigonal anti­prismatic for Cd2 with O_2_N trigonal faces. Selected geometrical parameters are given in Table 1 ▸.

**Figure 3:**
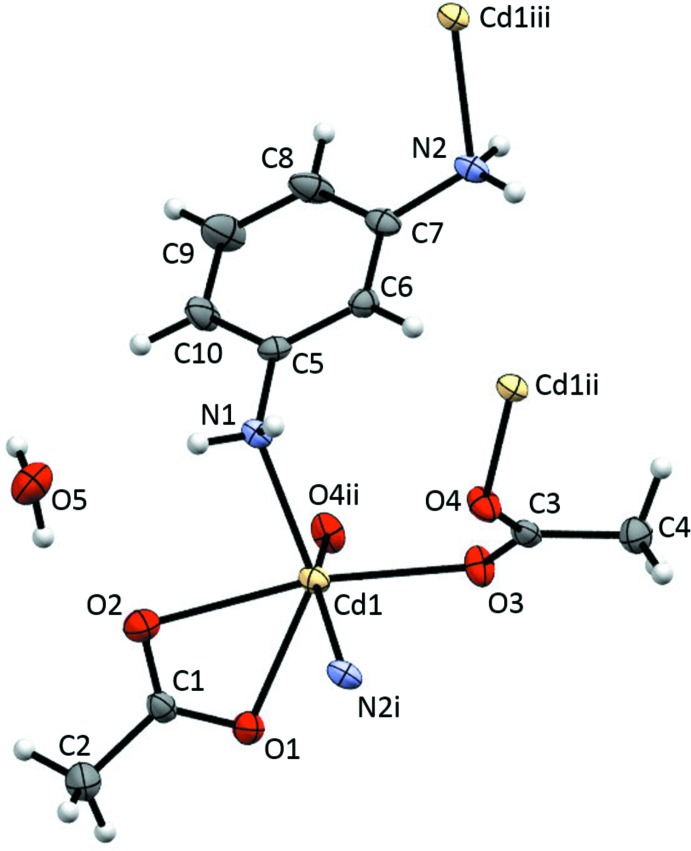
The atom-labeling scheme for (II). Anisotropic displacement parameters for non-H atoms are drawn at the 30% probability level. [Symmetry codes: (i) *x*, −*y* + 1, *z* + 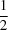; (ii) −*x* + 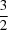, *y* − 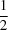, −*z* + 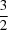; (iii) −*x* + 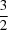, *y* + 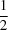, −*z* + 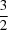.]

The N_2_O_4_ coordination geometry of (II) can be described as severely distorted trigonal anti­prismatic with bidentate acetate oxygen atoms and a κ^2^ *N*:*N*′ benzene-1,3-di­amine nitro­gen atom (O1O2N2^i^) forming one of the trigonal faces and two κ^2^ *O*:*O*′ acetate ligand oxygen atoms and a nitro­gen atom from a κ^2^ *N*:*N*′ benzene-1,3-di­amine (O3O4^ii^N1) forming the other trigonal face (see Fig. 2 ▸). The atom-labeling scheme is shown in Fig. 3 ▸. The twist angles are 53.71 (11), 22.56 (8) and 45.38 (13)° [average = 41 (16)°]. As seen in Table 2 ▸, the Cd—O bond lengths associated with the bidentate acetate ligand are shorter than those of the bridging, monodentate acetate ligands, as has been observed in other cadmium complexes (Wang *et al.*, 2011 ▸, 2013 ▸).

## Supra­molecular features   

**Figure 4:**
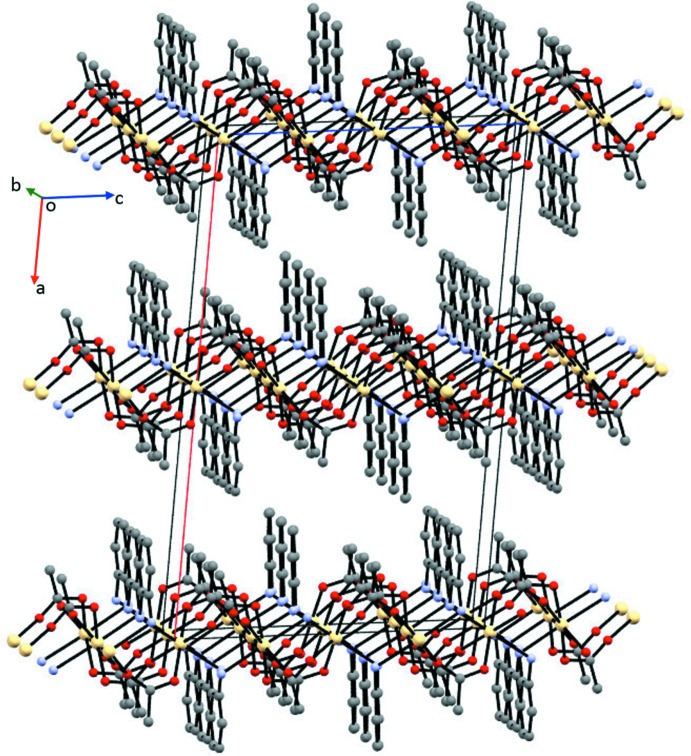
Packing diagram for (I) showing the two-dimensional network parallel to (100). All H atoms have been omitted for clarity.

As seen in Fig. 4 ▸, the supra­molecular architecture of (I) exhibits independent layers in the *bc* plane, which are repeated in the [100] direction. Extensive N—H⋯O hydrogen-bonding inter­actions exist (see Table 3 ▸), but none of them extend between the layers. Based on an analysis of the extended structure using the *SOLV* routine of *PLATON* (Spek, 2009 ▸), the unit cell contains no solvent-accessible voids.

**Figure 5:**
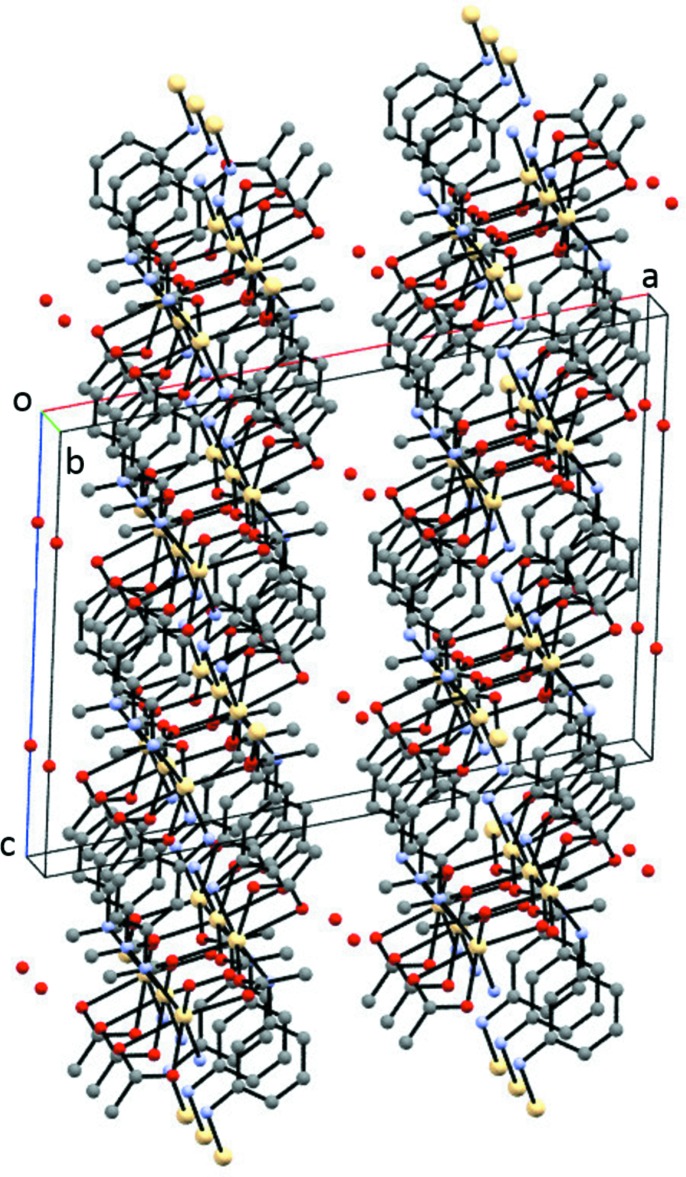
Packing diagram for (II) showing the layers parallel to (100). H atoms have been omitted for clarity.

**Figure 6:**
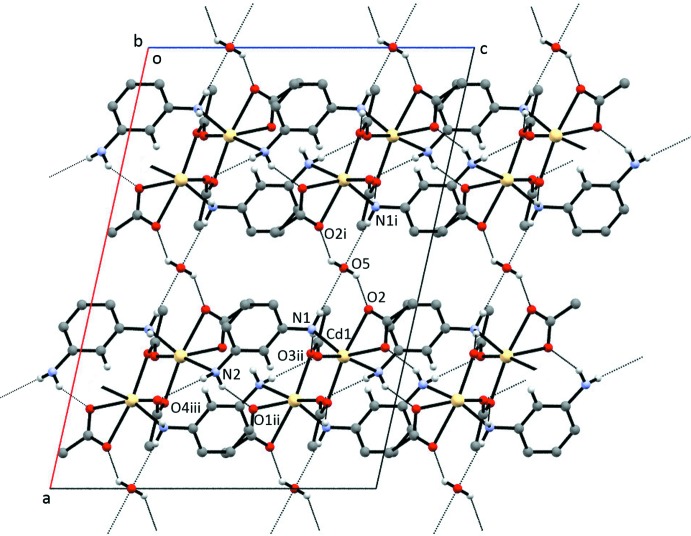
Partial packing diagram for (II) showing the hydrogen-bonded network. Only H atoms involved in the hydrogen-bonded network are shown. [Symmetry codes: (i) −*x* + 1, *y*, −*z* + 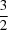; (ii) −*x* + 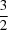, *y* + 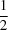, −*z* + 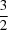; (iii)*x*, −*y* + 1, *z* − 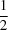.]

Compound (II) also exhibits a two-dimensional extended structure. Layers parallel to the *bc* plane and repeated in the [100] direction are observed as seen in Fig. 5 ▸. N—H⋯O(acetate) hydrogen bonds (Table 4 ▸) are present within the layers. The water of hydration sits on a crystallographically imposed twofold rotation axis and, as seen in Fig. 6 ▸, is involved in O—H⋯O and N—H⋯O hydrogen-bonding inter­actions (Table 4 ▸) that link adjacent layers.

## Database survey   

Examples of cadmium coordination polymers with carboxyl­ate ligands and that exhibit two-dimensional sheet structures have been reported (Li *et al.*, 2014 ▸; Gao *et al.*, 2004 ▸; Chen & Zhang, 2014 ▸; Zhang *et al.*, 2007 ▸; Liu & Xu, 2005 ▸; Song *et al.*, 2006 ▸; Kong *et al.*, 2008*a* ▸,*b* ▸; Xu *et al.*, 2013 ▸; Zhuo *et al.*, 2006 ▸). Cadmium is commonly observed with a trigonal–prismatic or trigonal–anti­prismatic coordination geometry, often with one or two capping ligands (Bygott *et al.*, 2007 ▸; Cherni *et al.*, 2012 ▸; Uçar *et al.*, 2004 ▸; Banerjee *et al.*, 2005 ▸; Keypour *et al.*, 2000 ▸). Coordination polymers with bridging benzene-1,2-di­amine ligands (Liang & Qu, 2008 ▸; Duff, 1968 ▸), bridging benzene-1,3-di­amine ligands (Chemli *et al.*, 2013 ▸), and bridging benzene-1,4-di­amine ligands (Liu *et al.*, 2012 ▸) have been reported

## Synthesis and crystallization   

### Preparation of (I)   

213 mg (0.924 mmole) cadmium acetate hydrate were dissolved in 10 mL of ethanol. With stirring, 204 mg (1.89 mmol) of benzene-1,2-di­amine were added and the resulting solution was refluxed for 2 h. A white precipitate formed, which was isolated by filtration and dried under vacuum. The yield was qu­anti­tative (310 mg). Selected IR bands (diamond anvil, cm^−1^): 3278 (*w*), 1532 (*s*), 1504 (*s*), 1405 (*s*). ^1^H NMR (400 MHz, dmso-*d*_6_, p.p.m.): 1.87 (*s*, 6H), 6.35 (*m*, 2H), 6.35 (*m*, 2H).

Single crystals were obtained by heating some of the product in *N*,*N*′-di­methyl­formamide and allowing the solution to slowly cool to room temperature. The crystal used for data collection was obtained by cutting a piece from a larger plate.

### Preparation of (II)   

230 mg (1.00 mmol) cadmium acetate hydrate were dissolved in 10 mL of ethanol. 217 mg (2.01 mmol) benzene-1,3-di­amine were added with stirring. The solution was gently refluxed for 2 h. After chilling the reaction mixture in an ice bath, the precipitate was filtered and dried under vacuum. A yield of 248 mg (71%) was obtained. Selected IR bands (diamond anvil, cm^−1^): 3425 (*b*), 3329 (*s*) 3328 (*b*), 3137 (*m*), 1520 (*s*), 1505 (*s*), 1400 (*s*). ^1^H NMR (400 MHz, dmso-*d*_6_, p.p.m.): 1.83 (*s*, 6H), 5.78 (*m*, 3H), 6.64 (*t*, 1H). ^13^C NMR (dmso-*d*_6_, p.p.m.): 22.1, 100.5, 103.6, 129.6, 149.5, 178.0.

Clear, brown needles suitable for X-ray analysis were obtained upon slow evaporation of an ethano­lic solution of the product. The crystals exhibit a melting range of 441–443 K with decomposition.

## Refinement   

Crystal data, data collection and structure refinement details are summarized in Table 5 ▸. For both (I) and (II), all hydrogen atoms were located in difference Fourier maps. The hydrogen atoms were refined using a riding model with a C—H distance of 0.98 Å for the methyl groups and 0.95 Å for the phenyl carbon atoms. The methyl hydrogen atom isotropic displacement parameters were set using the approximation *U*_iso_(H) = 1.5*U*_eq_(C). All other C—H hydrogen atom isotropic displacement parameters were set using the approximation *U*_iso_(H) = 1.2*U*_eq_(C). The N—H bond lengths were restrained to 0.88 Å in (I) and (II). The O—H bond length of the water of hydration in (II) was restrained to 0.84 Å and the H—O—H angle was restrained to 105°. *U*_iso_(H) was refined freely for the amine and water hydrogen atoms, except that for (II) the isotropic displacement parameters of the hydrogen atoms associated with N2 were restrained to be the same.

## Supplementary Material

Crystal structure: contains datablock(s) global, I, II. DOI: 10.1107/S2056989016017382/pj2037sup1.cif

Structure factors: contains datablock(s) I. DOI: 10.1107/S2056989016017382/pj2037Isup2.hkl

Supporting information file. DOI: 10.1107/S2056989016017382/pj2037Isup4.mol

Structure factors: contains datablock(s) II. DOI: 10.1107/S2056989016017382/pj2037IIsup3.hkl

Supporting information file. DOI: 10.1107/S2056989016017382/pj2037IIsup5.mol

CCDC references: 1512964, 1512963

Additional supporting information:  crystallographic information; 3D view; checkCIF report

## Figures and Tables

**Table 1:** Selected geometric parameters (Å, °) for (I)

Cd1—O3	2.323 (3)	Cd2—O4^i^	2.365 (3)
Cd1—O1	2.332 (3)	Cd2—O2	2.260 (3)
Cd1—N1	2.325 (4)	Cd2—N2^ii^	2.416 (4)
			
O3—Cd1—N1	84.79 (12)	O4^i^—Cd2—O4^iv^	80.73 (15)
O3—Cd1—O1	82.98 (11)	O2^iii^—Cd2—N2^ii^	79.40 (12)
N1—Cd1—O1	84.38 (12)	O2—Cd2—N2^ii^	115.81 (12)
O2^iii^—Cd2—O2	99.65 (17)	O4^i^—Cd2—N2^ii^	76.91 (12)
O2^iii^—Cd2—O4^i^	156.01 (10)	O4^iv^—Cd2—N2^ii^	85.97 (11)
O2—Cd2—O4^i^	93.98 (12)	N2^ii^—Cd2—N2^v^	157.5 (2)

Symmetry codes: (i) 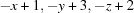; (ii) 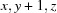; (iii) 
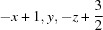
; (iv) 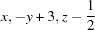; (v) 
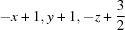
.

**Table 2:** Selected geometric parameters (Å, °) for (II)

Cd1—O3	2.275 (3)	Cd1—O1	2.374 (4)
Cd1—O4^i^	2.301 (3)	Cd1—N2^ii^	2.388 (4)
Cd1—N1	2.324 (4)	Cd1—O2	2.443 (4)
			
O3—Cd1—O4^i^	79.37 (11)	N1—Cd1—N2^ii^	101.86 (14)
O3—Cd1—N1	107.45 (13)	O1—Cd1—N2^ii^	84.00 (13)
O4^i^—Cd1—N1	99.25 (13)	O3—Cd1—O2	168.71 (11)
O3—Cd1—O1	114.63 (11)	O4^i^—Cd1—O2	98.78 (12)
O4^i^—Cd1—O1	85.82 (12)	N1—Cd1—O2	83.83 (13)
N1—Cd1—O1	137.80 (13)	O1—Cd1—O2	54.09 (11)
O3—Cd1—N2^ii^	86.63 (14)	N2^ii^—Cd1—O2	91.49 (13)
O4^i^—Cd1—N2^ii^	157.39 (13)		

Symmetry codes: (i) 
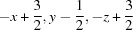
; (ii) 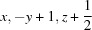.

**Table 3:** Hydrogen-bond geometry (Å, °) for (I)

*D*—H⋯*A*	*D*—H	H⋯*A*	*D*⋯*A*	*D*—H⋯*A*
N1—H1*A*⋯O2^iii^	0.86 (2)	2.34 (2)	3.175 (5)	163 (4)
N1—H1*B*⋯O4^vi^	0.91 (2)	2.21 (3)	3.003 (5)	146 (4)
N1—H1*B*⋯O4^vii^	0.91 (2)	2.38 (4)	3.029 (5)	128 (4)
N2—H2*A*⋯O3^viii^	0.86 (2)	2.30 (2)	3.111 (5)	158 (4)
N2—H2*B*⋯O3^vi^	0.86 (2)	2.64 (2)	3.458 (5)	161 (4)
N2—H2*B*⋯O4^vi^	0.86 (2)	2.55 (4)	2.973 (5)	111 (3)

Symmetry codes: (iii) 
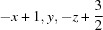
; (vi) 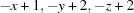; (vii) 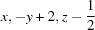; (viii) 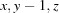.

**Table 4:** Hydrogen-bond geometry (Å, °) for (II)

*D*—H⋯*A*	*D*—H	H⋯*A*	*D*⋯*A*	*D*—H⋯*A*
O5—H5⋯O2	0.81 (2)	2.06 (4)	2.788 (5)	149 (7)
N1—H*N*1*A*⋯O5	0.86 (2)	2.34 (2)	3.183 (4)	166 (4)
N1—H*N*1*B*⋯O3^iii^	0.89 (2)	2.12 (2)	2.991 (5)	166 (5)
N2—H*N*2*A*⋯O4^iv^	0.87 (2)	2.32 (4)	3.012 (6)	137 (4)
N2—H*N*2*B*⋯O1^iii^	0.88 (2)	2.17 (3)	2.994 (5)	156 (5)

Symmetry codes: (iii) 
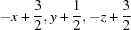
; (iv) 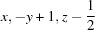.

**Table 5:** Experimental details

	(I)	(II)
Crystal data		
Chemical formula	[Cd_2_(C_2_H_3_O_2_)_4_(C_6_H_8_N_2_)_2_]	[Cd(C_2_H_3_O_2_)_2_(C_6_H_8_N_2_)]·0.5H_2_O
*M*_r_	338.63	695.28
Crystal system, space group	Monoclinic, *C*2/*c*	Monoclinic, *C*2/*c*
Temperature (K)	200	200
*a*, *b*, *c* (Å)	23.283 (3), 7.2399 (9), 14.2744 (16)	20.777 (6), 8.2374 (18), 15.002 (4)
β (°)	96.887 (4)	102.583 (9)
*V* (Å^3^)	2388.8 (5)	2505.9 (11)
*Z*	8	4
Radiation type	Mo *K*α	Mo *K*α
μ (mm^−1^)	1.83	1.75
Crystal size (mm)	0.40 × 0.40 × 0.05	0.40 × 0.08 × 0.08

Data collection		
Diffractometer	Bruker *SMART* X2S benchtop	Bruker *SMART* X2S benchtop
Absorption correction	Multi-scan (*SADABS*; Bruker, 2013 ▸)	Multi-scan (*SADABS*; Bruker, 2013 ▸)
*T*_min_, *T*_max_	0.53, 0.91	0.69, 0.87
No. of measured, independent and observed [*I* > 2σ(*I*)] reflections	14588, 2263, 1633	8942, 2443, 1859
*R*_int_	0.089	0.057
(sin θ/λ)_max_ (Å^−1^)	0.610	0.617

Refinement		
*R*[*F* ^2^ > 2σ(*F* ^2^)], *wR*(*F* ^2^), *S*	0.038, 0.109, 1.05	0.037, 0.095, 0.99
No. of reflections	2263	2443
No. of parameters	174	180
No. of restraints	4	6
H-atom treatment	H atoms treated by a mixture of independent and constrained refinement	H atoms treated by a mixture of independent and constrained refinement
Δρ_max_, Δρ_min_ (e Å^−3^)	1.14, −1.17	0.86, −1.02

Computer programs: *APEX2* and *SAINT* (Bruker, 2013 ▸), *SHELXS97* (Sheldrick, 2008 ▸), *SHELXL2014* (Sheldrick, 2015 ▸), *PLATON* (Spek, 2009 ▸), *Mercury* (Macrae *et al.*, 2006 ▸) and *publCIF* (Westrip, 2010 ▸).

## supplementary crystallographic information

### (I) Poly[tetra-µ2-acetato-κ8O:O'-bis(µ2-benzene-1,2-diamine-κ2N:N')dicadmium] Crystal data

**Table:**

[Cd_2_(C_2_H_3_O_2_)_4_(C_6_H_8_N_2_)_2_]	*F*(000) = 1344
*M**_r_* = 338.63	*D*_x_ = 1.883 Mg m^−^^3^
Monoclinic, *C*2/*c*	Mo *K*α radiation, λ = 0.71073 Å
*a* = 23.283 (3) Å	Cell parameters from 120 reflections
*b* = 7.2399 (9) Å	θ = 3.5–24.0°
*c* = 14.2744 (16) Å	µ = 1.83 mm^−^^1^
β = 96.887 (4)°	*T* = 200 K
*V* = 2388.8 (5) Å^3^	Plate, clear colourless
*Z* = 8	0.40 × 0.40 × 0.05 mm

### (I) Poly[tetra-µ2-acetato-κ8O:O'-bis(µ2-benzene-1,2-diamine-κ2N:N')dicadmium] Data collection

**Table:**

Bruker SMART X2S benchtop diffractometer	2263 independent reflections
Radiation source: sealed microfocus tube	1633 reflections with *I* > 2σ(*I*)
Doubly curved silicon crystal monochromator	*R*_int_ = 0.089
Detector resolution: 8.3330 pixels mm^-1^	θ_max_ = 25.7°, θ_min_ = 2.9°
ω scans	*h* = −28→27
Absorption correction: multi-scan (SADABS; Bruker, 2013)	*k* = −8→8
*T*_min_ = 0.53, *T*_max_ = 0.91	*l* = −17→16
14588 measured reflections	

### (I) Poly[tetra-µ2-acetato-κ8O:O'-bis(µ2-benzene-1,2-diamine-κ2N:N')dicadmium] Refinement

**Table:**

Refinement on *F*^2^	Primary atom site location: structure-invariant direct methods
Least-squares matrix: full	Secondary atom site location: difference Fourier map
*R*[*F*^2^ > 2σ(*F*^2^)] = 0.038	Hydrogen site location: mixed
*wR*(*F*^2^) = 0.109	H atoms treated by a mixture of independent and constrained refinement
*S* = 1.05	*w* = 1/[σ^2^(*F*_o_^2^) + (0.0496*P*)^2^ + 0.5909*P*] where *P* = (*F*_o_^2^ + 2*F*_c_^2^)/3
2263 reflections	(Δ/σ)_max_ < 0.001
174 parameters	Δρ_max_ = 1.14 e Å^−^^3^
4 restraints	Δρ_min_ = −1.17 e Å^−^^3^

### (I) Poly[tetra-µ2-acetato-κ8O:O'-bis(µ2-benzene-1,2-diamine-κ2N:N')dicadmium] Special details

**Table:**

Geometry. All esds (except the esd in the dihedral angle between two l.s. planes) are estimated using the full covariance matrix. The cell esds are taken into account individually in the estimation of esds in distances, angles and torsion angles; correlations between esds in cell parameters are only used when they are defined by crystal symmetry. An approximate (isotropic) treatment of cell esds is used for estimating esds involving l.s. planes.

### (I) Poly[tetra-µ2-acetato-κ8O:O'-bis(µ2-benzene-1,2-diamine-κ2N:N')dicadmium] Fractional atomic coordinates and isotropic or equivalent isotropic displacement parameters (Å^2^)

**Table:**

	*x*	*y*	*z*	*U*_iso_*/*U*_eq_	
Cd1	0.5	1.0	1.0	0.02022 (18)	
Cd2	0.5	1.48311 (6)	0.75	0.02246 (18)	
O1	0.53894 (13)	1.2329 (4)	0.91404 (19)	0.0280 (7)	
O2	0.57421 (14)	1.2817 (4)	0.7785 (2)	0.0341 (8)	
O3	0.43034 (13)	1.2226 (4)	1.01819 (19)	0.0287 (8)	
O4	0.44787 (12)	1.2680 (4)	1.17354 (18)	0.0275 (7)	
N1	0.44368 (16)	0.9351 (5)	0.8581 (2)	0.0220 (8)	
H1A	0.4354 (19)	1.040 (4)	0.831 (3)	0.033 (14)*	
H1B	0.4683 (16)	0.875 (6)	0.824 (3)	0.045 (15)*	
N2	0.44711 (17)	0.5481 (6)	0.8818 (2)	0.0226 (8)	
H2A	0.438 (2)	0.445 (4)	0.905 (3)	0.040 (15)*	
H2B	0.4719 (16)	0.612 (6)	0.917 (3)	0.044 (15)*	
C1	0.39101 (18)	0.8316 (6)	0.8544 (3)	0.0232 (10)	
C2	0.39284 (18)	0.6415 (6)	0.8678 (3)	0.0213 (10)	
C3	0.3425 (2)	0.5424 (7)	0.8632 (3)	0.0315 (12)	
H3	0.344	0.4118	0.8697	0.038*	
C4	0.2890 (2)	0.6318 (8)	0.8489 (3)	0.0409 (13)	
H4	0.2541	0.5629	0.8467	0.049*	
C5	0.2872 (2)	0.8209 (8)	0.8380 (3)	0.0403 (13)	
H5	0.251	0.8824	0.829	0.048*	
C6	0.3376 (2)	0.9219 (7)	0.8400 (3)	0.0326 (11)	
H6	0.336	1.052	0.8316	0.039*	
C7	0.57788 (19)	1.2169 (6)	0.8615 (3)	0.0267 (11)	
C8	0.6357 (3)	1.1326 (10)	0.8978 (4)	0.0650 (18)	
H8A	0.6648	1.2304	0.9089	0.098*	
H8B	0.6473	1.0454	0.8511	0.098*	
H8C	0.6324	1.0673	0.9571	0.098*	
C9	0.41522 (19)	1.2753 (6)	1.0965 (3)	0.0248 (10)	
C10	0.3540 (2)	1.3437 (8)	1.0964 (3)	0.0450 (14)	
H10A	0.3441	1.3462	1.1612	0.067*	
H10B	0.3274	1.2609	1.0581	0.067*	
H10C	0.3507	1.4686	1.0697	0.067*	

### (I) Poly[tetra-µ2-acetato-κ8O:O'-bis(µ2-benzene-1,2-diamine-κ2N:N')dicadmium] Atomic displacement parameters (Å^2^)

**Table:**

	*U*^11^	*U*^22^	*U*^33^	*U*^12^	*U*^13^	*U*^23^
Cd1	0.0250 (3)	0.0215 (3)	0.0140 (3)	−0.00045 (16)	0.0019 (2)	−0.00028 (16)
Cd2	0.0275 (3)	0.0228 (3)	0.0180 (3)	0	0.0066 (2)	0
O1	0.0330 (18)	0.0259 (18)	0.0270 (16)	0.0015 (14)	0.0107 (14)	0.0053 (14)
O2	0.047 (2)	0.034 (2)	0.0226 (18)	0.0065 (16)	0.0110 (14)	0.0081 (15)
O3	0.0396 (18)	0.0311 (19)	0.0166 (16)	0.0122 (15)	0.0083 (13)	0.0014 (14)
O4	0.0324 (18)	0.0324 (19)	0.0173 (16)	−0.0035 (15)	0.0010 (14)	−0.0029 (14)
N1	0.030 (2)	0.018 (2)	0.017 (2)	0.0000 (18)	0.0014 (16)	0.0001 (17)
N2	0.032 (2)	0.021 (2)	0.016 (2)	−0.0048 (19)	0.0047 (17)	0.0002 (18)
C1	0.032 (3)	0.028 (3)	0.010 (2)	−0.001 (2)	0.0050 (18)	−0.0021 (18)
C2	0.028 (2)	0.023 (2)	0.012 (2)	0.0015 (19)	0.0038 (18)	−0.0012 (18)
C3	0.039 (3)	0.030 (3)	0.026 (3)	−0.008 (2)	0.007 (2)	−0.004 (2)
C4	0.033 (3)	0.051 (4)	0.038 (3)	−0.006 (3)	0.004 (2)	−0.001 (3)
C5	0.030 (3)	0.049 (4)	0.041 (3)	0.011 (3)	0.004 (2)	0.003 (3)
C6	0.032 (3)	0.035 (3)	0.029 (3)	0.006 (2)	0.000 (2)	−0.002 (2)
C7	0.029 (3)	0.023 (3)	0.028 (3)	0.003 (2)	0.004 (2)	0.001 (2)
C8	0.065 (4)	0.066 (5)	0.065 (4)	0.004 (4)	0.013 (3)	0.002 (4)
C9	0.030 (2)	0.017 (2)	0.028 (3)	−0.0014 (19)	0.009 (2)	0.000 (2)
C10	0.036 (3)	0.063 (4)	0.036 (3)	0.016 (3)	0.006 (2)	−0.004 (3)

### (I) Poly[tetra-µ2-acetato-κ8O:O'-bis(µ2-benzene-1,2-diamine-κ2N:N')dicadmium] Geometric parameters (Å, º)

**Table:**

C1—C6	1.398 (6)	Cd1—O1^i^	2.332 (3)
C1—C2	1.390 (6)	Cd1—N1	2.325 (4)
C10—H10C	0.98	Cd1—N1^i^	2.325 (4)
C10—H10B	0.98	Cd2—O4^ii^	2.365 (3)
C10—H10A	0.98	Cd2—O4^iii^	2.365 (3)
C2—C3	1.369 (6)	Cd2—O2^iv^	2.260 (3)
C3—H3	0.95	Cd2—O2	2.260 (3)
C3—C4	1.398 (7)	Cd2—N2^v^	2.416 (4)
C4—H4	0.95	Cd2—N2^vi^	2.416 (4)
C4—C5	1.378 (7)	N1—H1B	0.907 (19)
C5—H5	0.95	N1—H1A	0.864 (19)
C5—C6	1.378 (7)	N1—C1	1.432 (5)
C6—H6	0.95	N2—H2B	0.857 (19)
C7—C8	1.512 (7)	N2—H2A	0.86 (2)
C8—H8C	0.98	N2—Cd2^vii^	2.415 (4)
C8—H8B	0.98	N2—C2	1.426 (6)
C8—H8A	0.98	O1—C7	1.250 (5)
C9—C10	1.509 (6)	O2—C7	1.267 (5)
Cd1—O3^i^	2.323 (3)	O3—C9	1.270 (5)
Cd1—O3	2.323 (3)	O4—Cd2^ii^	2.365 (3)
Cd1—O1	2.332 (3)	O4—C9	1.261 (5)
			
O3^i^—Cd1—O3	180.00 (13)	C2—N2—H2A	102 (3)
O3^i^—Cd1—N1	95.21 (12)	Cd2^vii^—N2—H2A	108 (3)
O3—Cd1—N1	84.79 (12)	C2—N2—H2B	110 (4)
O3^i^—Cd1—N1^i^	84.79 (12)	Cd2^vii^—N2—H2B	101 (3)
O3—Cd1—N1^i^	95.21 (12)	H2A—N2—H2B	115 (4)
N1—Cd1—N1^i^	180.0	C2—C1—C6	119.7 (4)
O3^i^—Cd1—O1	97.02 (11)	C2—C1—N1	120.1 (4)
O3—Cd1—O1	82.98 (11)	C6—C1—N1	120.2 (4)
N1—Cd1—O1	84.38 (12)	C3—C2—C1	120.1 (4)
N1^i^—Cd1—O1	95.62 (12)	C3—C2—N2	119.8 (4)
O3^i^—Cd1—O1^i^	82.98 (11)	C1—C2—N2	120.1 (4)
O3—Cd1—O1^i^	97.02 (11)	C2—C3—C4	120.5 (5)
N1—Cd1—O1^i^	95.62 (12)	C2—C3—H3	119.8
N1^i^—Cd1—O1^i^	84.38 (12)	C4—C3—H3	119.8
O1—Cd1—O1^i^	180.0	C5—C4—C3	119.4 (5)
O2^iv^—Cd2—O2	99.65 (17)	C5—C4—H4	120.3
O2^iv^—Cd2—O4^ii^	156.01 (10)	C3—C4—H4	120.3
O2—Cd2—O4^ii^	93.98 (12)	C4—C5—C6	120.7 (5)
O2^iv^—Cd2—O4^iii^	93.98 (11)	C4—C5—H5	119.6
O2—Cd2—O4^iii^	156.01 (10)	C6—C5—H5	119.6
O4^ii^—Cd2—O4^iii^	80.73 (15)	C5—C6—C1	119.6 (5)
O2^iv^—Cd2—N2^v^	79.40 (12)	C5—C6—H6	120.2
O2—Cd2—N2^v^	115.81 (12)	C1—C6—H6	120.2
O4^ii^—Cd2—N2^v^	76.91 (12)	O1—C7—O2	123.6 (4)
O4^iii^—Cd2—N2^v^	85.97 (11)	O1—C7—C8	120.8 (4)
O2^iv^—Cd2—N2^vi^	115.81 (12)	O2—C7—C8	115.3 (4)
O2—Cd2—N2^vi^	79.40 (12)	C7—C8—H8A	109.5
O4^ii^—Cd2—N2^vi^	85.97 (12)	C7—C8—H8B	109.5
O4^iii^—Cd2—N2^vi^	76.91 (12)	H8A—C8—H8B	109.5
N2^v^—Cd2—N2^vi^	157.5 (2)	C7—C8—H8C	109.5
C7—O1—Cd1	127.2 (3)	H8A—C8—H8C	109.5
C7—O2—Cd2	111.8 (3)	H8B—C8—H8C	109.5
C9—O3—Cd1	125.3 (3)	O4—C9—O3	123.6 (4)
C9—O4—Cd2^ii^	126.4 (3)	O4—C9—C10	119.1 (4)
C1—N1—Cd1	121.9 (2)	O3—C9—C10	117.3 (4)
C1—N1—H1A	107 (3)	C9—C10—H10A	109.5
Cd1—N1—H1A	107 (3)	C9—C10—H10B	109.5
C1—N1—H1B	109 (3)	H10A—C10—H10B	109.5
Cd1—N1—H1B	104 (3)	C9—C10—H10C	109.5
H1A—N1—H1B	107 (4)	H10A—C10—H10C	109.5
C2—N2—Cd2^vii^	120.6 (2)	H10B—C10—H10C	109.5
			
Cd1—N1—C1—C2	−75.0 (4)	C4—C5—C6—C1	−0.8 (7)
Cd1—N1—C1—C6	103.1 (4)	C2—C1—C6—C5	−0.9 (6)
C6—C1—C2—C3	2.6 (6)	N1—C1—C6—C5	−179.0 (4)
N1—C1—C2—C3	−179.3 (4)	Cd1—O1—C7—O2	131.3 (4)
C6—C1—C2—N2	179.5 (4)	Cd1—O1—C7—C8	−54.7 (6)
N1—C1—C2—N2	−2.4 (6)	Cd2—O2—C7—O1	13.3 (6)
Cd2^vii^—N2—C2—C3	99.3 (4)	Cd2—O2—C7—C8	−160.9 (4)
Cd2^vii^—N2—C2—C1	−77.6 (5)	Cd2^ii^—O4—C9—O3	100.8 (5)
C1—C2—C3—C4	−2.7 (6)	Cd2^ii^—O4—C9—C10	−81.4 (5)
N2—C2—C3—C4	−179.6 (4)	Cd1—O3—C9—O4	26.7 (6)
C2—C3—C4—C5	1.0 (7)	Cd1—O3—C9—C10	−151.1 (4)
C3—C4—C5—C6	0.8 (7)		

Symmetry codes: (i) −*x*+1, −*y*+2, −*z*+2; (ii) −*x*+1, −*y*+3, −*z*+2; (iii) *x*, −*y*+3, *z*−1/2; (iv) −*x*+1, *y*, −*z*+3/2; (v) *x*, *y*+1, *z*; (vi) −*x*+1, *y*+1, −*z*+3/2; (vii) *x*, *y*−1, *z*.

### (I) Poly[tetra-µ2-acetato-κ8O:O'-bis(µ2-benzene-1,2-diamine-κ2N:N')dicadmium] Hydrogen-bond geometry (Å, º)

**Table:**

*D*—H···*A*	*D*—H	H···*A*	*D*···*A*	*D*—H···*A*
N1—H1*A*···O2^iv^	0.86 (2)	2.34 (2)	3.175 (5)	163 (4)
N1—H1*B*···O4^i^	0.91 (2)	2.21 (3)	3.003 (5)	146 (4)
N1—H1*B*···O4^viii^	0.91 (2)	2.38 (4)	3.029 (5)	128 (4)
N2—H2*A*···O3^vii^	0.86 (2)	2.30 (2)	3.111 (5)	158 (4)
N2—H2*B*···O3^i^	0.86 (2)	2.64 (2)	3.458 (5)	161 (4)
N2—H2*B*···O4^i^	0.86 (2)	2.55 (4)	2.973 (5)	111 (3)
C8—H8*C*···O3^i^	0.98	2.61	3.295 (7)	127

Symmetry codes: (i) −*x*+1, −*y*+2, −*z*+2; (iv) −*x*+1, *y*, −*z*+3/2; (vii) *x*, *y*−1, *z*; (viii) *x*, −*y*+2, *z*−1/2.

### (II) Poly[[(µ2-acetato-κ2O:O')(acetato-κ2O,O')(µ2-benzene-1,3-diamine-κ2N:N')cadmium] hemihydrate] Crystal data

**Table:**

[Cd(C_2_H_3_O_2_)_2_(C_6_H_8_N_2_)]·0.5H_2_O	*F*(000) = 1384
*M**_r_* = 695.28	*D*_x_ = 1.843 Mg m^−^^3^
Monoclinic, *C*2/*c*	Mo *K*α radiation, λ = 0.71073 Å
*a* = 20.777 (6) Å	Cell parameters from 2588 reflections
*b* = 8.2374 (18) Å	θ = 2.7–23.5°
*c* = 15.002 (4) Å	µ = 1.75 mm^−^^1^
β = 102.583 (9)°	*T* = 200 K
*V* = 2505.9 (11) Å^3^	Needle, clear brown
*Z* = 4	0.40 × 0.08 × 0.08 mm

### (II) Poly[[(µ2-acetato-κ2O:O')(acetato-κ2O,O')(µ2-benzene-1,3-diamine-κ2N:N')cadmium] hemihydrate] Data collection

**Table:**

Bruker SMART X2S benchtop diffractometer	2443 independent reflections
Radiation source: sealed microfocus tube	1859 reflections with *I* > 2σ(*I*)
Doubly curved silicon crystal monochromator	*R*_int_ = 0.057
Detector resolution: 8.3330 pixels mm^-1^	θ_max_ = 26.0°, θ_min_ = 2.7°
ω scans	*h* = −25→25
Absorption correction: multi-scan (SADABS; Bruker, 2013)	*k* = −9→10
*T*_min_ = 0.69, *T*_max_ = 0.87	*l* = −18→11
8942 measured reflections	

### (II) Poly[[(µ2-acetato-κ2O:O')(acetato-κ2O,O')(µ2-benzene-1,3-diamine-κ2N:N')cadmium] hemihydrate] Refinement

**Table:**

Refinement on *F*^2^	6 restraints
Least-squares matrix: full	Hydrogen site location: mixed
*R*[*F*^2^ > 2σ(*F*^2^)] = 0.037	H atoms treated by a mixture of independent and constrained refinement
*wR*(*F*^2^) = 0.095	*w* = 1/[σ^2^(*F*_o_^2^) + (0.0489*P*)^2^] where *P* = (*F*_o_^2^ + 2*F*_c_^2^)/3
*S* = 0.99	(Δ/σ)_max_ = 0.001
2443 reflections	Δρ_max_ = 0.86 e Å^−^^3^
180 parameters	Δρ_min_ = −1.02 e Å^−^^3^

### (II) Poly[[(µ2-acetato-κ2O:O')(acetato-κ2O,O')(µ2-benzene-1,3-diamine-κ2N:N')cadmium] hemihydrate] Special details

**Table:**

Geometry. All esds (except the esd in the dihedral angle between two l.s. planes) are estimated using the full covariance matrix. The cell esds are taken into account individually in the estimation of esds in distances, angles and torsion angles; correlations between esds in cell parameters are only used when they are defined by crystal symmetry. An approximate (isotropic) treatment of cell esds is used for estimating esds involving l.s. planes.

### (II) Poly[[(µ2-acetato-κ2O:O')(acetato-κ2O,O')(µ2-benzene-1,3-diamine-κ2N:N')cadmium] hemihydrate] Fractional atomic coordinates and isotropic or equivalent isotropic displacement parameters (Å^2^)

**Table:**

	*x*	*y*	*z*	*U*_iso_*/*U*_eq_	
Cd1	0.69991 (2)	0.34064 (4)	0.80989 (2)	0.02831 (14)	
O1	0.68026 (16)	0.1764 (4)	0.9313 (2)	0.0381 (9)	
O2	0.59435 (17)	0.3087 (4)	0.8546 (2)	0.0436 (9)	
O3	0.80578 (15)	0.3370 (3)	0.7917 (3)	0.0357 (8)	
O4	0.79978 (15)	0.6013 (4)	0.7691 (2)	0.0375 (8)	
O5	0.5	0.5121 (7)	0.75	0.0581 (16)	
H5	0.517 (3)	0.455 (4)	0.792 (2)	0.09 (2)*	
N1	0.63907 (19)	0.5002 (5)	0.6938 (3)	0.0281 (9)	
HN1A	0.6021 (14)	0.522 (5)	0.708 (3)	0.039 (15)*	
HN1B	0.649 (2)	0.605 (3)	0.701 (3)	0.039 (14)*	
N2	0.7361 (2)	0.4639 (5)	0.4277 (3)	0.0328 (9)	
HN2A	0.750 (2)	0.394 (5)	0.393 (3)	0.044 (11)*	
HN2B	0.7682 (17)	0.526 (5)	0.457 (3)	0.044 (11)*	
C1	0.6204 (2)	0.2190 (6)	0.9196 (3)	0.0316 (11)	
C2	0.5810 (3)	0.1613 (6)	0.9855 (4)	0.0553 (16)	
H2A	0.5764	0.2496	1.0275	0.083*	
H2B	0.6034	0.0692	1.0204	0.083*	
H2C	0.5372	0.1272	0.952	0.083*	
C3	0.8315 (2)	0.4712 (5)	0.7778 (3)	0.0283 (10)	
C4	0.9037 (2)	0.4701 (6)	0.7760 (4)	0.0423 (13)	
H4A	0.9093	0.5044	0.7156	0.064*	
H4B	0.9213	0.3602	0.7888	0.064*	
H4C	0.9275	0.5451	0.8224	0.064*	
C5	0.6364 (2)	0.4410 (5)	0.6031 (3)	0.0276 (10)	
C6	0.6875 (2)	0.4739 (5)	0.5613 (3)	0.0292 (11)	
H6	0.7238	0.5375	0.5921	0.035*	
C7	0.6866 (2)	0.4152 (5)	0.4748 (3)	0.0314 (11)	
C8	0.6335 (3)	0.3195 (6)	0.4305 (4)	0.0463 (14)	
H8	0.6327	0.2769	0.3714	0.056*	
C9	0.5833 (3)	0.2881 (7)	0.4720 (4)	0.0573 (17)	
H9	0.5473	0.2237	0.4413	0.069*	
C10	0.5832 (3)	0.3481 (5)	0.5588 (4)	0.0436 (14)	
H10	0.5475	0.3258	0.5871	0.052*	

### (II) Poly[[(µ2-acetato-κ2O:O')(acetato-κ2O,O')(µ2-benzene-1,3-diamine-κ2N:N')cadmium] hemihydrate] Atomic displacement parameters (Å^2^)

**Table:**

	*U*^11^	*U*^22^	*U*^33^	*U*^12^	*U*^13^	*U*^23^
Cd1	0.0331 (2)	0.0280 (2)	0.0254 (2)	0.00293 (14)	0.00988 (14)	−0.00140 (14)
O1	0.0347 (19)	0.045 (2)	0.036 (2)	0.0083 (15)	0.0105 (16)	0.0041 (15)
O2	0.043 (2)	0.049 (2)	0.038 (2)	0.0063 (16)	0.0066 (17)	0.0087 (16)
O3	0.0333 (17)	0.0217 (17)	0.056 (2)	−0.0019 (13)	0.0175 (17)	0.0015 (15)
O4	0.0414 (19)	0.0292 (17)	0.047 (2)	0.0059 (15)	0.0204 (17)	0.0114 (15)
O5	0.044 (3)	0.065 (4)	0.058 (4)	0	−0.004 (3)	0
N1	0.032 (2)	0.029 (2)	0.026 (2)	−0.0001 (19)	0.0113 (17)	0.0008 (17)
N2	0.041 (2)	0.036 (2)	0.024 (2)	−0.0003 (19)	0.0143 (18)	−0.0007 (18)
C1	0.033 (3)	0.035 (3)	0.028 (3)	−0.006 (2)	0.011 (2)	−0.005 (2)
C2	0.041 (3)	0.073 (4)	0.054 (4)	0.004 (3)	0.014 (3)	0.014 (3)
C3	0.033 (2)	0.033 (3)	0.021 (2)	−0.001 (2)	0.0095 (19)	−0.0018 (18)
C4	0.036 (3)	0.045 (3)	0.045 (3)	−0.006 (2)	0.007 (2)	0.004 (2)
C5	0.034 (2)	0.025 (2)	0.022 (2)	0.0006 (19)	0.002 (2)	0.0044 (17)
C6	0.029 (2)	0.030 (2)	0.027 (2)	−0.0059 (19)	0.002 (2)	0.0020 (19)
C7	0.041 (3)	0.025 (2)	0.029 (3)	0.004 (2)	0.011 (2)	0.0054 (19)
C8	0.070 (4)	0.043 (3)	0.027 (3)	−0.020 (3)	0.013 (3)	−0.008 (2)
C9	0.070 (4)	0.064 (4)	0.037 (3)	−0.041 (3)	0.009 (3)	−0.014 (3)
C10	0.044 (3)	0.052 (3)	0.035 (3)	−0.025 (2)	0.012 (2)	−0.001 (2)

### (II) Poly[[(µ2-acetato-κ2O:O')(acetato-κ2O,O')(µ2-benzene-1,3-diamine-κ2N:N')cadmium] hemihydrate] Geometric parameters (Å, º)

**Table:**

Cd1—O3	2.275 (3)	C1—C2	1.492 (8)
Cd1—O4^i^	2.301 (3)	C2—H2A	0.98
Cd1—N1	2.324 (4)	C2—H2B	0.98
Cd1—O1	2.374 (4)	C2—H2C	0.98
Cd1—N2^ii^	2.388 (4)	C3—C4	1.506 (6)
Cd1—O2	2.443 (4)	C4—H4A	0.98
O1—C1	1.268 (5)	C4—H4B	0.98
O2—C1	1.249 (5)	C4—H4C	0.98
O3—C3	1.265 (5)	C5—C6	1.374 (6)
O4—C3	1.250 (5)	C5—C10	1.388 (6)
O4—Cd1^iii^	2.301 (3)	C6—C7	1.381 (6)
O5—H5	0.808 (18)	C6—H6	0.95
N1—C5	1.435 (6)	C7—C8	1.401 (6)
N1—HN1A	0.859 (19)	C8—C9	1.350 (8)
N1—HN1B	0.886 (19)	C8—H8	0.95
N2—C7	1.425 (6)	C9—C10	1.394 (8)
N2—Cd1^iv^	2.388 (4)	C9—H9	0.95
N2—HN2A	0.870 (19)	C10—H10	0.95
N2—HN2B	0.877 (19)		
			
O3—Cd1—O4^i^	79.37 (11)	C1—C2—H2A	109.5
O3—Cd1—N1	107.45 (13)	C1—C2—H2B	109.5
O4^i^—Cd1—N1	99.25 (13)	H2A—C2—H2B	109.5
O3—Cd1—O1	114.63 (11)	C1—C2—H2C	109.5
O4^i^—Cd1—O1	85.82 (12)	H2A—C2—H2C	109.5
N1—Cd1—O1	137.80 (13)	H2B—C2—H2C	109.5
O3—Cd1—N2^ii^	86.63 (14)	O4—C3—O3	122.3 (4)
O4^i^—Cd1—N2^ii^	157.39 (13)	O4—C3—C4	120.5 (4)
N1—Cd1—N2^ii^	101.86 (14)	O3—C3—C4	117.1 (4)
O1—Cd1—N2^ii^	84.00 (13)	C3—C4—H4A	109.5
O3—Cd1—O2	168.71 (11)	C3—C4—H4B	109.5
O4^i^—Cd1—O2	98.78 (12)	H4A—C4—H4B	109.5
N1—Cd1—O2	83.83 (13)	C3—C4—H4C	109.5
O1—Cd1—O2	54.09 (11)	H4A—C4—H4C	109.5
N2^ii^—Cd1—O2	91.49 (13)	H4B—C4—H4C	109.5
C1—O1—Cd1	93.7 (3)	C6—C5—C10	120.3 (5)
C1—O2—Cd1	91.0 (3)	C6—C5—N1	119.4 (4)
C3—O3—Cd1	117.6 (3)	C10—C5—N1	120.3 (5)
C3—O4—Cd1^iii^	136.7 (3)	C5—C6—C7	120.4 (4)
C5—N1—Cd1	114.9 (3)	C5—C6—H6	119.8
C5—N1—HN1A	117 (3)	C7—C6—H6	119.8
Cd1—N1—HN1A	108 (3)	C6—C7—C8	119.4 (5)
C5—N1—HN1B	114 (3)	C6—C7—N2	120.2 (4)
Cd1—N1—HN1B	113 (3)	C8—C7—N2	120.1 (5)
HN1A—N1—HN1B	88 (4)	C9—C8—C7	119.8 (5)
C7—N2—Cd1^iv^	114.3 (3)	C9—C8—H8	120.1
C7—N2—HN2A	119 (3)	C7—C8—H8	120.1
Cd1^iv^—N2—HN2A	96 (3)	C8—C9—C10	121.4 (5)
C7—N2—HN2B	118 (4)	C8—C9—H9	119.3
Cd1^iv^—N2—HN2B	94 (3)	C10—C9—H9	119.3
HN2A—N2—HN2B	111 (5)	C5—C10—C9	118.7 (5)
O2—C1—O1	121.0 (5)	C5—C10—H10	120.6
O2—C1—C2	120.0 (4)	C9—C10—H10	120.6
O1—C1—C2	119.0 (4)		
			
Cd1—O2—C1—O1	−4.6 (4)	N1—C5—C6—C7	178.6 (4)
Cd1—O2—C1—C2	174.9 (4)	C5—C6—C7—C8	−1.0 (7)
Cd1—O1—C1—O2	4.8 (5)	C5—C6—C7—N2	173.2 (4)
Cd1—O1—C1—C2	−174.8 (4)	Cd1^iv^—N2—C7—C6	−103.9 (4)
Cd1^iii^—O4—C3—O3	−148.4 (4)	Cd1^iv^—N2—C7—C8	70.2 (5)
Cd1^iii^—O4—C3—C4	34.3 (6)	C6—C7—C8—C9	1.1 (8)
Cd1—O3—C3—O4	−3.8 (6)	N2—C7—C8—C9	−173.1 (5)
Cd1—O3—C3—C4	173.5 (3)	C7—C8—C9—C10	−0.4 (9)
Cd1—N1—C5—C6	−82.6 (4)	C6—C5—C10—C9	0.6 (7)
Cd1—N1—C5—C10	95.9 (4)	N1—C5—C10—C9	−177.9 (5)
C10—C5—C6—C7	0.1 (6)	C8—C9—C10—C5	−0.5 (9)

Symmetry codes: (i) −*x*+3/2, *y*−1/2, −*z*+3/2; (ii) *x*, −*y*+1, *z*+1/2; (iii) −*x*+3/2, *y*+1/2, −*z*+3/2; (iv) *x*, −*y*+1, *z*−1/2.

### (II) Poly[[(µ2-acetato-κ2O:O')(acetato-κ2O,O')(µ2-benzene-1,3-diamine-κ2N:N')cadmium] hemihydrate] Hydrogen-bond geometry (Å, º)

**Table:**

*D*—H···*A*	*D*—H	H···*A*	*D*···*A*	*D*—H···*A*
O5—H5···O2	0.81 (2)	2.06 (4)	2.788 (5)	149 (7)
N1—H*N*1*A*···O5	0.86 (2)	2.34 (2)	3.183 (4)	166 (4)
N1—H*N*1*B*···O3^iii^	0.89 (2)	2.12 (2)	2.991 (5)	166 (5)
N2—H*N*2*A*···O4^iv^	0.87 (2)	2.32 (4)	3.012 (6)	137 (4)
N2—H*N*2*B*···O1^iii^	0.88 (2)	2.17 (3)	2.994 (5)	156 (5)
C6—H6···O1^iii^	0.95	2.39	3.195 (5)	142

Symmetry codes: (iii) −*x*+3/2, *y*+1/2, −*z*+3/2; (iv) *x*, −*y*+1, *z*−1/2.
